# miR-29a-3p suppresses cell proliferation and migration by downregulating IGF1R in hepatocellular carcinoma

**DOI:** 10.18632/oncotarget.21246

**Published:** 2017-09-23

**Authors:** Xiao Wang, Shasha Liu, Ling Cao, Tengfei Zhang, Dongli Yue, Liping Wang, Yu Ping, Qianyi He, Chaoqi Zhang, Meng Wang, Xinfeng Chen, Qun Gao, Dan Wang, Zhen Zhang, Fei Wang, Li Yang, Jieyao Li, Lan Huang, Bin Zhang, Yi Zhang

**Affiliations:** ^1^ Biotherapy Center, The First Affiliated Hospital of Zhengzhou University, Zhengzhou, Henan 450052, P.R. China; ^2^ Department of Oncology, The First Affiliated Hospital of Zhengzhou University, Zhengzhou, Henan 450052, P.R. China; ^3^ School of Life Sciences, Zhengzhou University, Zhengzhou, Henan 450052, P.R. China; ^4^ Department of Hematology/Oncology, School of Medicine, Northwestern University, Chicago, IL 60611, USA; ^5^ Key Laboratory for Tumor Immunology and Biotherapy of Henan Province, Zhengzhou, Henan 450052, P.R. China

**Keywords:** miR-29a-3p, IGF1R, hepatocellular carcinoma, proliferation, CCL5

## Abstract

Hepatocellular carcinoma (HCC), the most common primary tumor of the liver, has a poor prognosis and rapid progression. MicroRNAs (miRNAs) play important roles in carcinogenesis and tumor progression. Insulin-like growth factor 1 receptor (IGF1R) is a transmembrane heterotetrameric protein that has been reported to promote transformation to malignancy and cancer cell proliferation and survival. In this study, we found that the expression of miR-29a-3p was downregulated in HCC patients, resulting in poor survival rates. Contrastingly, the overexpression of miR-29a-3p significantly inhibited proliferation and migration in HepG2 cells. miR-29a-3p directly targeted IGF1R and down-regulated its expression. Moreover, knockdown of IGF1R led to the increased production of chemokine ligand 5 (CCL5). In tumor lesions, the local expression of CCL5 negatively affected the expression of IGF1R. Transwell analysis showed that CCL5 was important for the chemotactic movement of CD8^+^ T lymphocytes. The expression of CCL5 in HCC tissues positively correlated with the expression of CD8^+^ T lymphocyte surface marker, CD8. Patients with high CCL5 expression exhibited better survival. Our results revealed that miR-29a-3p is a tumor suppressor gene that acts by directly repressing the oncogene IGF1R, which takes part in immunoregulation in tumor microenvironments in HCC, implying that miR-29a-3p could be a potential target for HCC treatment.

## INTRODUCTION

Hepatocellular carcinoma (HCC) is a primary neoplasm of the liver and the fourth most common cause of cancer-related death worldwide [1]. Its onset characteristics are not apparent, and has a high degree of malignancy with poor prognosis and rapid progression. Since effectively diagnosing HCC at its early stage is particularly difficult, only about 10–20% of the patients with HCC are eligible for surgical treatment and even then, some of these patients suffer from the recurring tumors [2, 3]. Therefore, it is necessary to develop effective markers for the diagnosis, prognosis, and treatment of patients with this disease.

miRNAs are a class of short, evolutionarily conserved, endogenous, single-stranded, non-coding RNA molecules which regulate gene expression at the post-transcriptional level [4]. miRNAs play a major role in many fundamentally important biological processes including cell cycle, differentiation, development, and metabolism [5, 6]. They can work both as tumor suppressors and as oncogenes [7–10]. This dual role as oncomiRs and tumor suppressors has stimulated multiple studies on miRNAs and cancers [11]. Emerging evidence indicates that some tumor-specific miRNAs are widely downregulated or upregulated in HCC and closely associated with the occurrence and development of HCC [12–15]. A recent study has shown that there are 17 miRNAs with high expression and 9 miRNAs with low expression in HCC [16].

miR-29a, currently one of the most interesting miRNA families in humans, has been shown to be silenced or downregulated in many different types of cancers such as prostate cancer, renal cell carcinoma, gastric cancer, and pancreatic cancer. It has also been shown to mediate either tumor suppressive or oncogenic functions in distinct malignancies [17–20]. These contrary findings suggested that miR-29a was involved in tumor progression. Therefore, its specific biological function and regulation in HCC needs to be elucidated.

In this study, we found that miR-29a-3p was a regulator of insulin-like growth factor 1 receptor (IGF1R) in HCC. High expression of miR-29a-3p in HCC tissues provided better prognosis. Mechanistically, miR-29a-3p inhibited HCC proliferation, migration *in vitro* and tumor growth *in vivo***.** Therefore, restoration of miR-29a-3p expression in HCC cells could reduce cell proliferation and suppress cell migration and tumor formation. Meanwhile, we found that knockdown of IGF1R could give rise to the increase of CCL5 secretion. In tumor lesions, the local expression of CCL5 was negatively associated with the expression of IGF1R. CCL5 served as a key chemokine to recruit CD8^+^ T lymphocytes into HCC tissue. The expression of CCL5 in HCC tissues was positively correlated with the local expression of CD8^+^ T lymphocyte. Patients with high CCL5-expression exhibited better survival rates.

## RESULTS

### miR-29a-3p expression level was downregulated and associated with better prognosis in HCC

**Figure 1:**
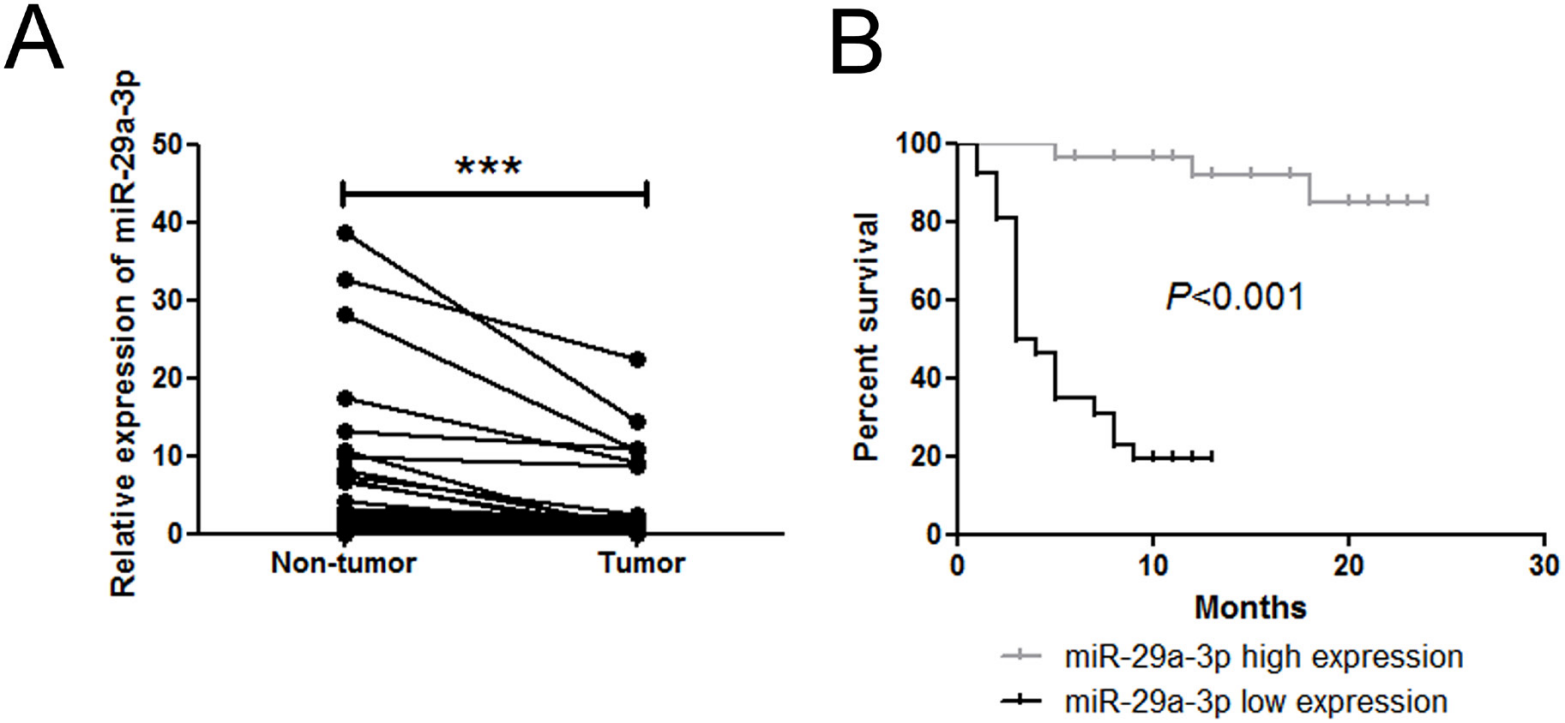
miR-29a-3p expression level in 62 patient samples **(A)** QRT-PCR analysis of miR-29a-3p expression in 62 HCC tissue samples and their corresponding adjacent non-tumorous liver samples (***P < 0.001). The expression of miR-29a-3p was normalized to U6 snRNA. **(B)** Kaplan-Meier analysis associated with overall survival for low and high miR-29a-3p expression (*P* < 0.001).

To study the expression of miR-29a-3p in HCC, we first examined the expression pattern of miR-29a-3p in 62 paired HCC samples and adjacent non-tumor tissue samples by quantitative real-time PCR (QRT-PCR). Compared with non-tumor samples, HCC samples exhibited lower levels of miR-29a-3p expression (Figure 1A). Additionally, miR-29a-3p expression level was correlated with better prognosis (Figure 1B), implicating that miR-29a-3p might play a role in inhibiting HCC tumorigenicity.

### miR-29a-3p could repress growth and migration of HCC cells

**Figure 2:**
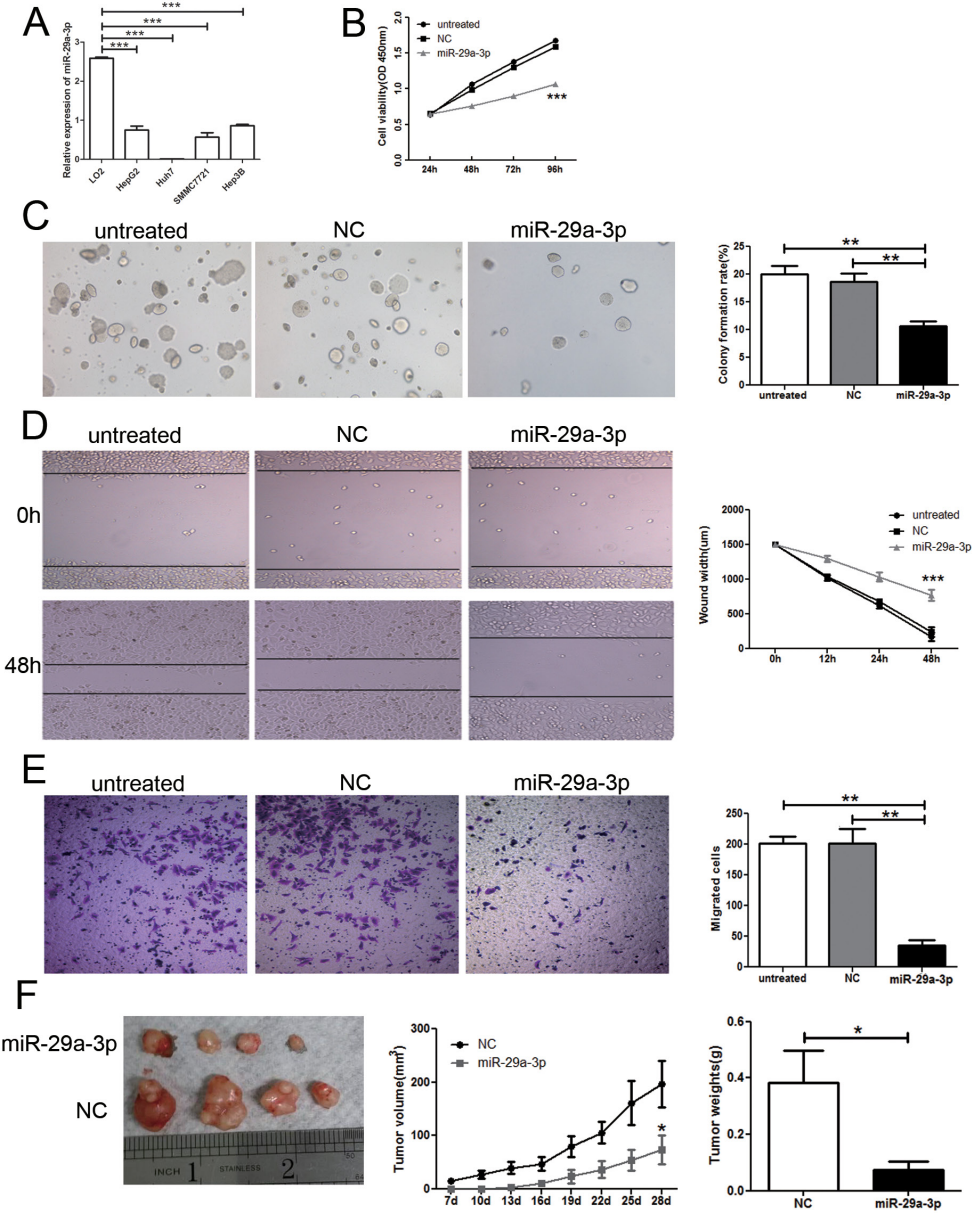
Overexpression of miR-29a-3p inhibited cancer cell growth and migration *in vitro* and *in vivo* **(A)** QRT-PCR analysis of miR-29a-3p expression in normal human hepatic cell line (LO2) and HCC cells lines (SMMC-7721, Hep3B, HepG2, and Huh 7). **(B)** Proliferation ability test by CCK8 assay of HepG2 cells after transfection with miR-29a-3p mimics, negative control (NC) or no transfection (untreated). **(C)** Colony formation assay and statistical results in HepG2 cells after transfection with miR-29a-3p mimics, NC or untreated. **(D)** Wound healing assay of HepG2 cells after transfection with the miR-29a-3p mimics, NC or untreated. **(E)** Transwell migration assay of HepG2 cells after transfection with the miR-29a-3p mimics, NC or untreated. **(F)** Functional test of miR-29a-3p *in vivo* and statistical results. (*P < 0.05, **P < 0.01, ***P < 0.001).

To understand the role of miR-29a-3p, firstly, we tested the miR-29a-3p expression in normal human hepatic cell line (LO2) and HCC cell lines (SMMC-7721, Hep3B, HepG2, and Huh 7) by QRT-PCR. miR-29a-3p was reduced in all the HCC cell lines when compared to LO2 (Figure 2A). To address the function of miR-29a-3p in HCC, we examined its effects on cell growth by CCK8 assay and colony formation assay. HepG2 cells were transfected with either negative control or miR-29a-3p mimics. Reduced viability was observed in cells with miR-29a-3p overexpression compared to non-transfected cells and cells transfected with negative control (Figure 2B). Moreover, cells with miR-29a-3p overexpression formed fewer colonies than the control cells (Figure 2C).

To evaluate the migratory potential of HepG2 cells transfected with miR-29a-3p, wound healing assay was performed *in vitro*. The results revealed that tumor cells with negative control rapidly closed the scratch wounds compared to the miR-29a-3p mimics (*P*<0.001) (Figure 2D). Moreover, the wounds of HepG2 cells transfected with miR-29a-3p were still open, while the wounds in HepG2 cells transfected with negative control had almost closed at 48 h, which suggested that HepG2 cells with miR-29a-3p transfection had lower migratory potential in relation to HepG2 cells with negative control transfection. We further performed *in vitro* Transwell migration assay to investigate the effect of miR-29a-3p on the migrative ability of HepG2 cells *in vitro*. miR-29a-3p overexpression resulted in decreased HepG2 cell invasion rate compared with the control cells (*P*< 0.05) (Figure 2E). To investigate the effects of miR-29a-3p on tumorigenesis *in vivo*, HepG2 cells transfected with miR-29a-3p mimics or negative control were injected subcutaneously into nude mice to initiate tumor formation. Four weeks later, large tumors were observed in the negative control groups, while the tumor volume was still minimal in those mice transplanted with the miR-29a-3p overexpression cells (Figure 2F). At the end of the experiments, the tumors were isolated (Figure 2F) and weighed. Tumors from the nude mice transfected with miR-29a-3p mimics weighed significantly less than the control mice (Figure 2F). These results were consistent with the antiproliferation function of miR-29a-3p *in vitro* and indicated that miR-29a-3p overexpression elicited a strong anti-tumor effect in HCC *in vivo*. These results clearly indicated that miR-29a-3p overexpression could inhibit growth and migration of HepG2 cells.

### miR-29a-3p directly targeted IGF1R

**Figure 3:**
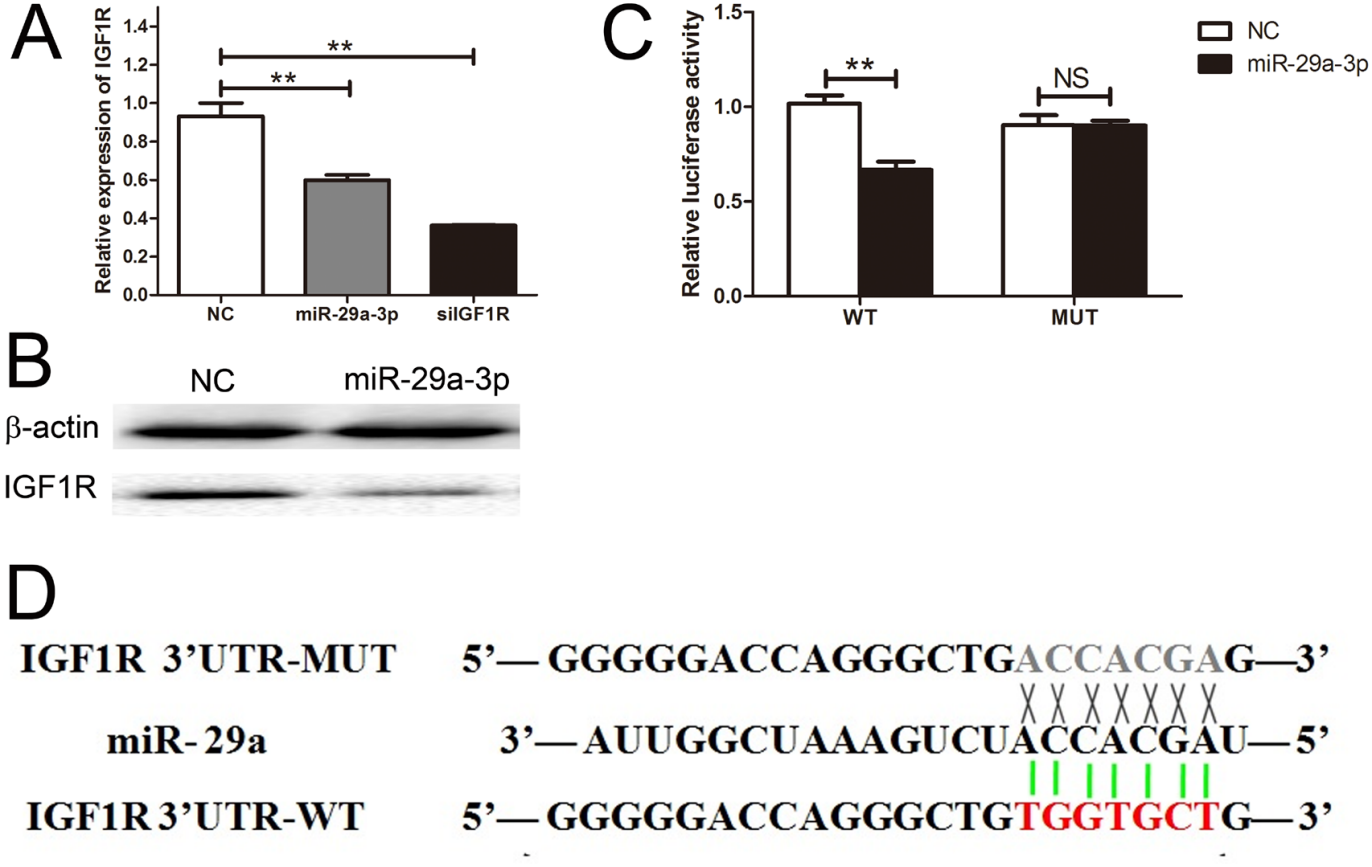
IGF1R was a direct target gene of miR-29a-3p **(A)** The relative expression of IGF1R after transfection with miR-29a-3p mimics, NC and siIGF1R. **(B)** Western blot analysis of IGF1R expression in HepG2 cells transfected with miR-29a-3p mimics and NC. **(C)** The analysis of the relative luciferase activities of IGF1R-WT and IGF1R-MUT in HepG2 cells. **(D)** The sequences of miR-29a-3p banding sites within the human IGF1R 3' UTR and schematic reporter constructs.

We identified IGF1R mRNA as one of the putative miR-29a-3p targets by using the miRNA database, Targetscan. To validate this prediction, we transfected HepG2 cells with miR-29a-3p mimics, negative control, and siIGF1R, and investigated the IGF1R expression level with QRT-PCR. The result showed that the IGF1R expression level of HepG2 cells after transfection with siIGF1R was lower than the other two groups (Figure 3A). Meanwhile, we further tested the IGF1R expression level by western blot assay and found that IGF1R expression level negatively correlated with miR-29a-3p (Figure 3B). In line with the above results, we identified the binding sites of miR-29a-3p in IGF1R 3'UTR with the Luciferase Reporter Assay (Figure 3C, 3D), showing that IGF1R was directly regulated by miR-29a-3p.

### The expression of IGF1R was upregulated in HCC resulting in poor prognosis

**Figure 4:**
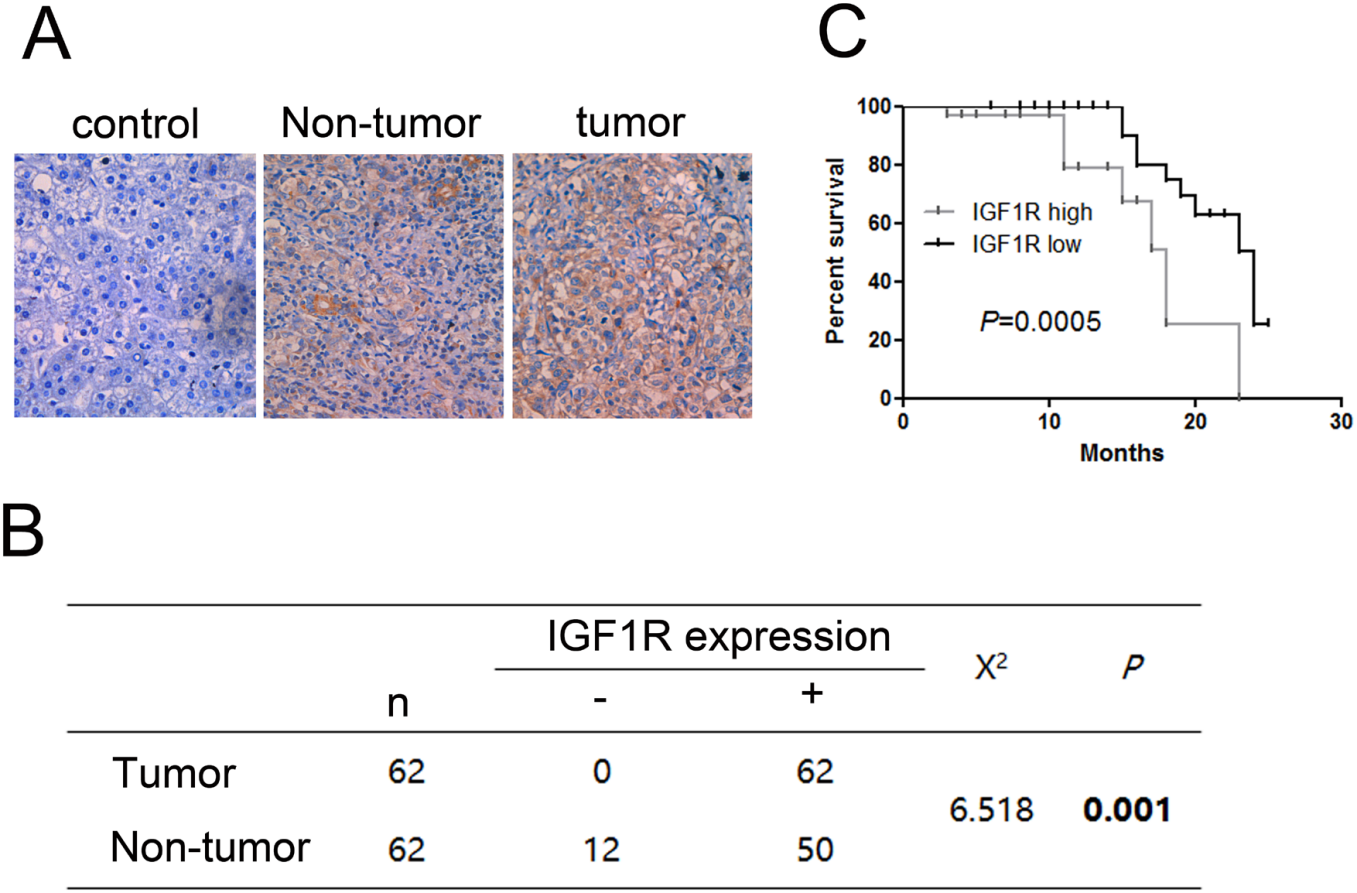
Immunohistochemistry test of IGF1R in 62 HCC tissues and their corresponding adjacent non-tumorous livers **(A)** Expression of IGF1R in paired tumor samples and their corresponding adjacent non-tumorous livers. **(B)** Chi-square test of the 62 patients for the paired tissues. Each group was shown by the distribution of IHC staining scores. **(C)** Kaplan-Meier analysis associated with overall survival for low and high expression of IGF1R (*P* = 0.0005). Original magnification: 400×.

We measured the protein expression level of IGF1R in 62paired HCC samples and adjacent non-tumor tissue samples by IHC. We found significant differences between the tumor and the adjacent non-tumor tissue samples. The expression of IGF1R is higher in HCC than in adjacent non-tumor tissue samples (Figure 4A, 4B). In addition, the expression level of IGF1R was associated with poor prognosis (Figure 4C).

### Knockdown of IGF1R led to the increase of CCL5 secretion

**Figure 5:**
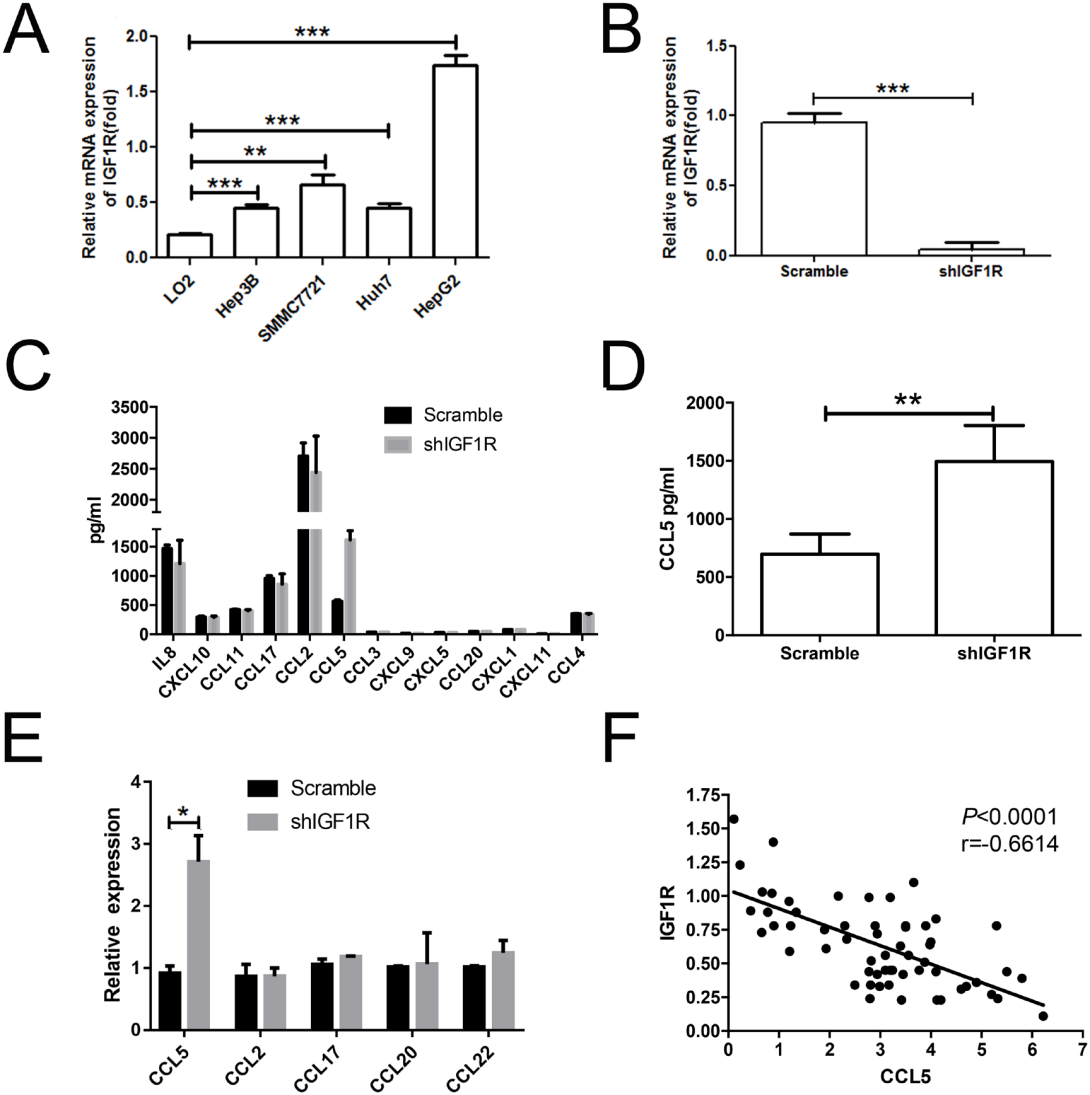
Knockdown of IGF1R led to the increase of CCL5 secretion **(A)** Real-time PCR analysis of IGF1R expression in LO2, SMMC-7721, Hep3B, HepG2, and Huh7 cell lines. **(B)** The mRNA level of IGF1R was verified in sorted HepG2 cells after transfection. **(C)** Results of scramble shRNA and shIGF1R HepG2 cells supernatants analyzed by multiplex assay in relation to chemokines. **(D)** The protein level of CCL5 in sorted HepG2 cells was assessed by using ELISA. **(E)** The level of CCL5 in sorted HepG2 cells was assessed by RT-PCR. **(F)** Association between the expression of IGF1R and the intensities of CCL5 in tumor lesions. (*P < 0.05, **P < 0.01, ***P < 0.001).

Chemokines, secreted by the tumor cells from primary tumors or metastatic sites or by the normal cells, recruited and/or locally activated immune cells [21, 22]. The IGF1R has been associated with chemokine production [23, 24]. Therefore, we tested the IGF1R expression in normal human hepatic cell line (LO2) and HCC cell lines (SMMC-7721, Hep3B, HepG2, and Huh 7) by QRT-PCR. IGF1R increased in all the HCC cell lines when compared to LO2 (Figure 5A). Furthermore, we used a lentiviral system to generate a stable IGF1R knockdown cell line. Two short hairpin RNAs (shRNAs) designated as scramble and shIGF1R were specially designed and constructed. After transfection for 72 h, the stably transfected HepG2 cells were sorted by flow cytometry. The cells were cultured for 2 weeks and the purities of sorted scramble and shIGF1R HepG2 cells were 98.8% and 97.2%, respectively. Real-time PCR was used to confirm the knockdown efficiency of IGF1R. The level of IGF1R mRNA expression significantly reduced in shIGF1R cells when compared to the scramble cells (Figure 5B). The above results demonstrated that the expression of IGF1R could be downregulated by shRNAs specifically and effectively.

We analyzed the supernatant of shIGF1R-HepG2 cells and scramble-HepG2 cells by multiplex assay. CCL5 expression changed most significantly in response to IGF1R knockdown (Figure 5C). We validated the level of CCL5 by ELISA and RT-PCR. The results showed that the level of CCL5 was higher in shIGF1R than scramble shRNA (Figure 5D, 5E), indicating that IGF1R could inhibit CCL5 production. We then performed an RT-PCR assay. In tumor lesions, the local expression of CCL5 was negatively associated with the expression of the IGF1R (r =-0.6614, *p***<** 0.0001) (Figure 5F).

### CCL5 was important for the chemotactic migration of CD8^+^ T lymphocytes

**Figure 6:**
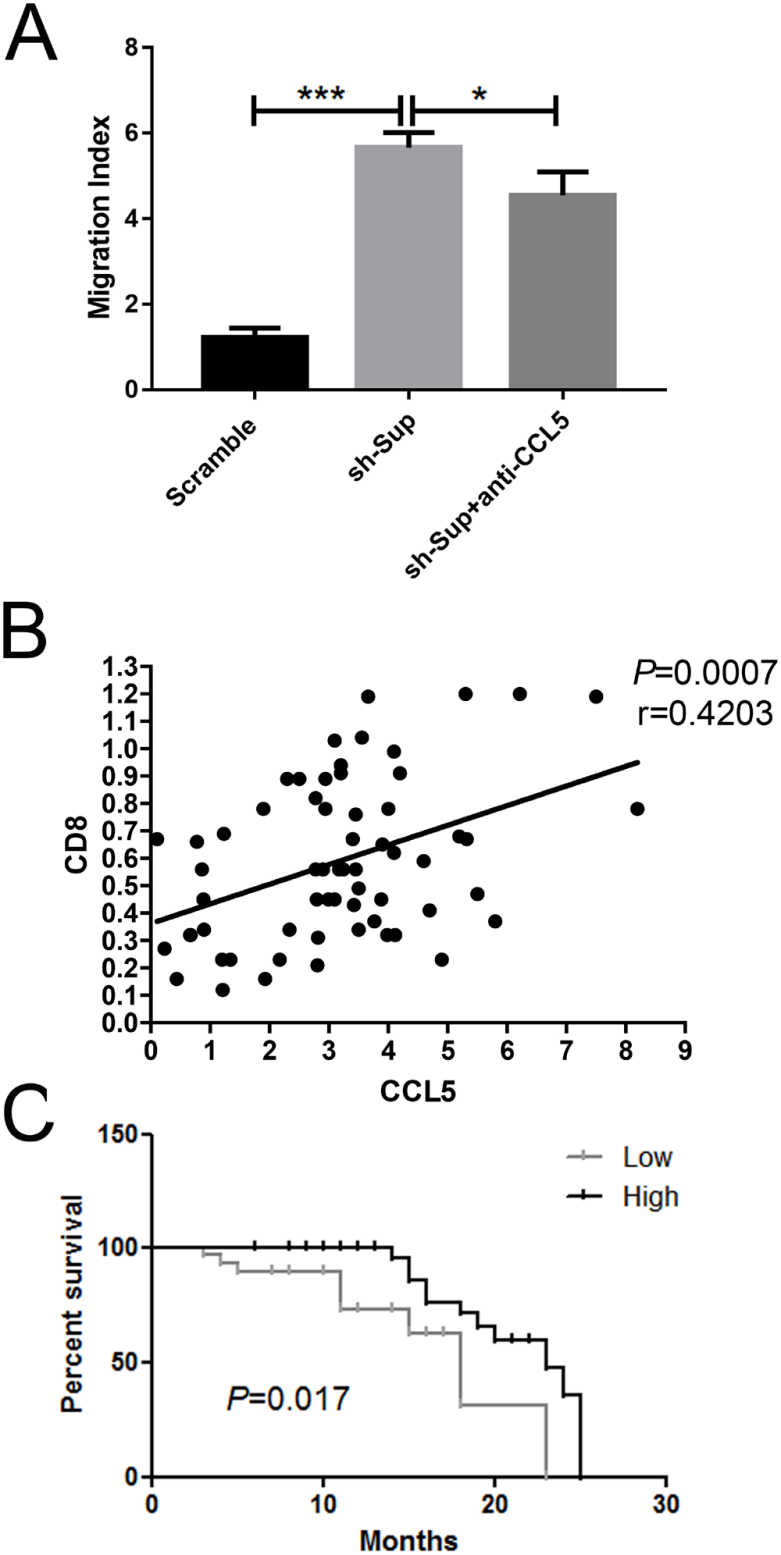
CCL5 recruits CD8+ T lymphocytes *in vitro* **(A)** The migration cells of CD8^+^ were counted using a limited 60-s analysis on a flow cytometer after the treatment with scramble HepG2 cells/shIGF1R HepG2 cells/anti-CCL5. **(B)** Association between the expression of CD8^+^ effector T lymphocyte markers CD8 and the intensities of CCL5 in tumor lesions (n=62). **(C)** Kaplan-Meier analysis associated with overall survival for low and high expressions of CCL5 (n=62; *P* =0.017; *P < 0.05, **P < 0.01, ***P < 0.001).

Previous reports have been shown that CCL5 could recruit CD8^+^ T cells [25, 26]. To ascertain the regulatory effects of CCL5 on CD8^+^ T lymphocyte migration, Transwell assay was performed. Scramble-HepG2 cells and shIGF1R-HepG2 cells were cultured in DMEM supplemented with 10% fetal bovine serum (FBS) and the supernatants were collected 48 h later. Additionally, CD8^+^ T lymphocytes were magnetically isolated from fresh peripheral blood mononuclear cells (PBMCs). The purity of T lymphocytes used was greater than 90% (data not shown). Compared to scramble-HepG2 cell supernatants, the supernatants derived from shIGF1R-HepG2 cells robustly enhanced the movement of T lymphocytes (*p* < 0.001) (Figure 6A). However, the neutralizing antibody for CCL5 significantly hampered the migration induced by shIGF1R-HepG2 cells supernatant (*p* CCL5 < 0.05) (Figure 6A). These results indicated that CCL5 was important in attracting CD8^+^ T lymphocytes towards HCC, suggesting that IGF1R, as an oncogene, could suppress CD8^+^ T cell recruitment to tumor environments by inhibiting CCL5 production.

To check whether CCL5 exerts an impact on the tumoral accumulation of CD8^+^ T lymphocytes, we performed an RT-PCR assay. In tumor lesions, the local expression of CCL5 was positively associated with the expression of the CD8^+^ T lymphocyte marker CD8 (r=0.4203, *p*=0.0007) (Figure 6B). Altogether, these data demonstrated that CCL5 predicts the recruitment and retention of CD8^+^ T lymphocytes in HCC.

Lastly, we also examined the expression level of CCL5 in 62 HCC samples and found that the CCL5 expression level was correlated with better prognosis (Figure 6C). The results indicated that CCL5 might play a role in HCC tumorigenicity.

## DISCUSSION

HCC is one of the most common cancers in the world and has an extremely poor prognosis [27, 28]. Its underlying molecular mechanism remains largely unknown even today. Accumulating evidence has revealed that dysregulation of miRNAs may contribute to its tumorigenesis [29]. The miR-29 family, which includes miR-29a, miR-29b, and miR-29c, is involved in cell cycle, apoptosis, and tumorigenesis [30, 31]. However, little is known about the role of miR-29a-3p during HCC development.

In this study, we analyzed the expression of miR-29a-3p in 62 HCC patients and HCC cell lines and found that miR-29a-3p expression was downregulated in HCC cell lines and HCC tissues when compared with the paired adjacent non-tumor tissues. miR-29a-3p regulated several hallmarks of cancer including cell growth, migration, and tumor formation. Our findings suggest that miR-29a-3p has a fundamental role in HCC tumorigenesis and cancer cell proliferation and invasion.

Previous studies have shown that miR-29a may act as a potential suppressor miRNA [32–34]. For example, miR-29a was downregulated in cervical squamous cell carcinoma tissues and was correlated with its progression by inhibiting cervical cancer cell migration and invasion [32]. However, a study demonstrated that miR-29a was upregulated in colorectal cancer tissue [35]. Furthermore, miR-29a is also involved in Alzheimer’s disease [36], HIV-1 replication [37], liver fibrosis [38], and HCV replication [39]. Therefore, the underlying mechanism of miR-29a-3p in HCC is still unknown.

To elucidate the role of miR-29a-3p in the progression of HCC, cell transfection was carried out. The overexpression of miR-29a-3p significantly suppressed HCC cell proliferation and migration. Moreover, we found that miR-29a-3p was negatively correlated with tumor size and provided a better prognosis. These data indicated that a high expression of miR-29a-3p might suppress tumor development and progression in HCC. These results also implied that miR-29a-3p might act as a tumor-suppressor whose downregulation contributed to the progression of HCC.

IGF1R is a transmembrane tyrosine protein receptor with tyrosine kinase activity and frequently overexpresses in various cancers including colorectal cancer. It functions as a key oncogene in the development and maintenance of cancers [40]. In this study, we identified IGF1R as a direct target gene of miR-29a-3p in HepG2 cells using bioinformatic prediction, Dual-Luciferase Reporter Assay and Western blot. We also showed that the overexpression of miR-29a-3p inhibited IGF1R protein expression. IGF1R expression was obviously upregulated in HCC tissues, and its overexpression was negatively correlated with the prognosis of HCC. These findings suggested that IGF1R was the target gene of miR-29a-3p.

Immunoregulation plays an important role in the occurrence and development of HCC. Various chemokines are involved in this process. In the present study, we showed that the level of CCL5 was significantly increased by shRNA-induced IGF1R silencing, and in tumor lesions, the local expression of CCL5 was negatively associated with the expression of the IGF1R, suggesting that IGF1R took part in immunoregulation in the tumor microenvironment. However, the underlying mechanism that elucidated the IGF1R upregulation by CCL5 secretion in HCC is still unknown.

CCL5 is one of the C-C chemokines and a potent chemoattractant for T lymphocytes, monocytes, natural killer cells, and eosinophils [41, 42]. In this study, we found that the expression of CCL5 in HCC tissues was positively correlated with the local expression of CD8^+^ T lymphocyte marker CD8. These observations strongly indicated that CCL5 is involved in the local recruitment and retention of CD8^+^ T lymphocytes to HCC lesions. CCL5 was positively correlated with HCC patient survival; that is, patients with high CCL5-expression had better survival. The impact of CCL5 on survival is likely associated with the accumulation of CD8^+^ T lymphocytes. Furthermore, CCL5 recruited CD8^+^ T lymphocytes into HCC tissue, suggesting that CCL5 could improve the curative efficacy of such adoptive therapy.

## MATERIALS AND METHODS

### Ethics statement

The study protocol was approved by the ethics committee of the First Affiliated Hospital of Zhengzhou University (Henan), and all HCC patients provided written informed consents regarding the use of clinical specimens for the study.

### Clinical specimens and cell culture

Sixty-two HCC tissue samples were collected from patients who underwent hepatectomy as treatment of HCC at the First Affiliated Hospital of Zhengzhou University, Henan. Information pertaining to the clinicopathological parameters was also available. Liver cancer cell lines (HepG2, Hep3B, Huh-7, and SMMC-7721) were provided by the cell bank of the Chinese Academy of Sciences and the normal human hepatic cell line (LO2) was preserved in our laboratory and maintained in RPMI-1640 supplemented with 10% FBS (GIBCO, USA), 100 U/ml of penicillin, and 100 μg/ml of streptomycin at 37°C in a 5% CO_2_ incubator.

### Cell transfection

The miR-29a-3p mimics and the negative control were synthesized by GenePharma (Shanghai, China) and transfected into the HepG2 cells to a final oligonucleotide concentration of 20 nmol/L. All cell transfections were introduced by Lipofectamine^®^ 3000 (Invitrogen Life Technologies, USA) according to the manufacturer’s instructions. For each cell transfection, three replicates were performed.

### RNA extraction, reverse transcription, and quantitative real-time PCR

Total RNA was extracted from HepG2, Hep3B, Huh-7, SMMC-7721, and LO2 cells and 62 tissue specimens by using TRIzol reagent (Invitrogen Life Technologies, USA) according to the manufacturer's instructions. The RNA was quantified by assessing its absorbance at 260 nm. The cDNA was synthesized from 2 μg of total RNA using miScript II RT Kit (QIAGEN, Germany). Stem-loop RT primers were used for the reverse transcription of miRNAs. The cDNA was used as a template to detect the expression of miR-29a in HepG2, Hep3B, Huh-7, SMMC-7721, and LO2 cells and tissue specimens. Real-time quantitative PCR (QRT-PCR) detection of genes in cell lines was performed using FastStart Essential DNA Green Master (Roche, USA) according to the manufacturer’s instructions. U6 was used as an internal control. The data were analyzed by 2^-ΔΔCt^. Primer sequences for QRT-PCR are shown in Table 1. Experiments were conducted in duplicates.

**Table 1:** The sequences of primers used

Gene	Sequence
miR-29a-3p	Forward primer: AGCACCAUCUGAAAUCGGUUA
	universal primer: GTGCAGGGTCCGAGGT
U6	Forward primer: CTCGCTTCGGCAGCACA
	Reverse primer: AACGCTTCACGAATTTGCGT
IGF1R	Forward primer: CCCGGCATCTTACTACATGG
	Reverse primer: GAAGGAACTGAAGCATTGG
GAPDH	Sense: 5′-GGAGCCAAAAGGGTCATCATCTC-3′
	Anti-sense: 5′-GAGGGGCCATCCACAGTCTTCT-3′
CD8	Sense: 5′-CGCTGTCAGATCCCCTTTGT-3′
	Anti-sense: 5′-GAGGAAGGACCCTCTCCCTT-3′
CCL5	Sense: 5′-CAGTCGTCTTTGTCACCCGA-3′
	Anti-sense: 5′-TGTAACTGCTGCTGTGTGGT-3′

The concentration and purity of RNA was detected using the NanoDrop 2000 (Thermo Scientific). The first strand cDNA was synthesized from 1 μg of total RNA using PrimeScript RT Reagent Kit with gDNA Eraser (TaKaRa, Japan) following which the genes of interest were tested. Primers used for this experiment are listed in Table 1. Quantitative real-time PCR was performed using SYBR Premix Ex Taq II (TaKaRa, Japan) in the Agilent Mx3005P qPCR system. Each experiment was performed in triplicate. Glyceraldehyde-3-phosphate dehydrogenase (GAPDH) was used as an endogenous control for normalization.

### CCK8 assay

Harvested cells were seeded into 96-well plates at 1.5 × 10^3^ per well (n = 4 for each time point) in a final volume of 100 μL. Cells were cultured for 24, 48, 72, and 96 h after transfection. CCK-8 (10 μL) was added into each well, and the absorbance was measured at 450 nm after incubation for 2 h at 37°C.

### Colony formation assay

HepG2 cells were transfected with either miR-29a-3p mimics or negative control. Cells were suspended in complete medium containing 0.6% low-melting-point agar (Sigma) and then applied to the top of a 1.2% agar/complete medium layer in 6-well plates. Two weeks later, colonies were formed and counted. The method is in agreement with those reported in previous studies [43]. All the experiments were performed in triplicates.

### Wound healing and migration assays

Cell migration was assessed by wound healing and Transwell assays. Cells were seeded into 6-well culture dishes and cultured to 100% confluence. Wounds were generated in the cell monolayer using a plastic pipette tip. The cells were then rinsed with PBS and cultured for another 24 h. The wound closure process was observed and photographed under a microscope. For migration assays, 1.5 × 10^5^ cells in serum-free media were added into the upper chamber (BD Bioscience). The lower chamber was filled with RPMI1640 with 10% FBS. After 24 h of incubation, the cells remaining on the upper surface of the membrane was removed, whereas the cells that had migrated through the membrane were stained with 20% methanol and 0.2% crystal violet, imaged, and counted under a microscope (Olympus, Tokyo, Japan).

### Animal experiments

All animal experiments were approved by the Ethical Committee on Animal Experiments at the University of Zhengzhou Animal Care Committee, Henan, China. For tumor growth assays, HepG2 cells infected with either the miR-29a-3p mimics or the negative control were injected subcutaneously into the right scapulas of nude mice (5-week-old BALB/c-nude, 4 per group, 2.0 × 10^6^ cells for each mouse). The mice were observed over 4 weeks for tumor formation. The tumor volume (V) was monitored every 3 days and calculated using the formula: V = 0.5 × length × width^2^.

### Dual-Luciferase reporter assay

We constructed reporter plasmids containing wild-type Luc-IGF1R (WT) and mutant Luc-IGF1R 3' UTR. miR-29a-3p mimics were synthesized by GenePharma Co., Ltd (Shanghai, China). We tested the luciferase activity of the indicated cells by Dual-Luciferase Reporter Assay System (Promega, Madison, WI, USA) 24 h after transfection, according to the manufacturer’s instructions.

### Western blot analysis

All the tested cells were lysed using lysis buffer and total proteins were quantified by the BCA assay. Protein samples from cells were separated by SDS-PAGE using a 10% (w/v) acrylamide gel and were transferred to a PVDF membrane. The bands were visualized by an enhanced chemiluminescence kit after incubation with primary and secondary antibodies. The intensities of individual bands were analyzed by densitometry using IMAGEL (National Institutes of Health Software, Bethesda, MD, USA) and normalized to the ß-actin level.

### Immunochemistry analysis

Sections (4-μm thick) were cut from paraffin blocks consecutively, deparaffinized in xylene, and rehydrated in a graded alcohol series. According to the manufacturer’s protocol, immunochemistry staining and the previously listed analytic methods were in agreement with a previous study [44]. Anti-IGF1R (Abcam, 1:1000) was used in the experiments.

### Lentiviral generation and cell sorting

A stable knockdown of IGF1R in HepG2 cells by shRNA was achieved using pGLV-H1-GFP+Puro vector plasmid purchased from GenePharma (GenePharma, Shanghai, China). For viral transductions, 500 μL of the pGLV-H1-shControl or pGLV-H1-shIGF1R lentiviruses were transfected with HepG2 cells, and the expression of IGF1R was confirmed by real-time RT-PCR. After 48 h, the transfected cells were sorted by flow cytometry (Beckman MoFlo XDP, Brea, CA, USA) according to the expression of the green fluorescent protein (GFP).

### Multiplex assay

Scramble shRNA and shIGF1R HepG2 cells were cultured in complete medium in 24-well plates for 3 days. All the supernatants were collected for the multi-analyte flow assay kit (BioLegend, USA) according to the manufacturer’s instructions. This allowed for the simultaneous quantification of 13 human chemokines.

### Enzyme-linked immunosorbent assay

Scramble shRNA and shIGF1R HepG2 cells were cultured in complete medium in 24-well plates for 3 days. All the supernatants were collected for enzyme-linked immunosorbent assay (ELISA), which was used to assess the levels of CCL5 (B210285; BioLegend, USA) in the supernatant according to the manufacturer’s instructions.

### Chemotaxis assay

The chemotactic migration of CD8+ T lymphocytes was evaluated in 24-well plates with 5-μm pore size polycarbonate filters (Corning Inc, Coring, NY, USA). First, PBMCs were isolated from heparinized blood samples by Ficoll-Paque Plus density centrifugation. Next, 600 μL of Scramble-HepG2 cells/shIGF1R-HepG2 cells supernatants were placed into the lower chambers of the Transwell plates. Anti-CCL5 (1 μg/mL) antibodies were added as per manufacturer’s instructions. Purified CD8^+^ T lymphocytes from PBMCs (purity > 90%) were counted. Then 5 × 10^5^ CD8^+^ T lymphocytes were added into the upper chambers and incubated at 37°C in a 5% CO_2_ atmosphere for 2 h. Cells in the bottom chambers were counted using a limited 60s analysis on a flow cytometer.

### Statistical analysis

Data are expressed as mean ± SD and analyzed using the student's t-test. Paired t-test was used for paired samples. Non-parametric test was performed for samples of non-normal distribution. The Spearman test was adapted to determine the correlation between the chemokine gene and CD8^+^ T lymphocyte marker. Overall survival curves were plotted according to the Kaplan-Meier method. Statistical analyses were conducted with SPSS 17.0 software. *P*<0.05 was considered to indicate a statistically significant difference.

## Abbreviations

HCC: Hepatocellular carcinoma
miRNAs: microRNAs
IGF1R: Insulin-like growth factor 1 receptor
CCL5: chemokine ligand 5
PBMC: peripheral blood mononuclear cell
