# Correction: miR-29a-3p suppresses cell proliferation and migration by downregulating IGF1R in hepatocellular carcinoma

**DOI:** 10.18632/oncotarget.28398

**Published:** 2023-05-19

**Authors:** Xiao Wang, Shasha Liu, Ling Cao, Tengfei Zhang, Dongli Yue, Liping Wang, Yu Ping, Qianyi He, Chaoqi Zhang, Meng Wang, Xinfeng Chen, Qun Gao, Dan Wang, Zhen Zhang, Fei Wang, Li Yang, Jieyao Li, Lan Huang, Bin Zhang, Yi Zhang

**Affiliations:** ^1^Biotherapy Center, The First Affiliated Hospital of Zhengzhou University, Zhengzhou, Henan 450052, P.R. China; ^2^Department of Oncology, The First Affiliated Hospital of Zhengzhou University, Zhengzhou, Henan 450052, P.R. China; ^3^School of Life Sciences, Zhengzhou University, Zhengzhou, Henan 450052, P.R. China; ^4^Department of Hematology/Oncology, School of Medicine, Northwestern University, Chicago, IL 60611, USA; ^5^Key Laboratory for Tumor Immunology and Biotherapy of Henan Province, Zhengzhou, Henan 450052, P.R. China

**This article has been corrected:** In Figure 2D, parts of the NC and miR-29a-3p panels are accidental duplicates. The corrected Figure 2, obtained using the original data, is shown below. The authors declare that these corrections do not change the results or conclusions of this paper.

Original article: Oncotarget. 2017; 8:86592–86603. 86592-86603. https://doi.org/10.18632/oncotarget.21246

**Figure 2:**
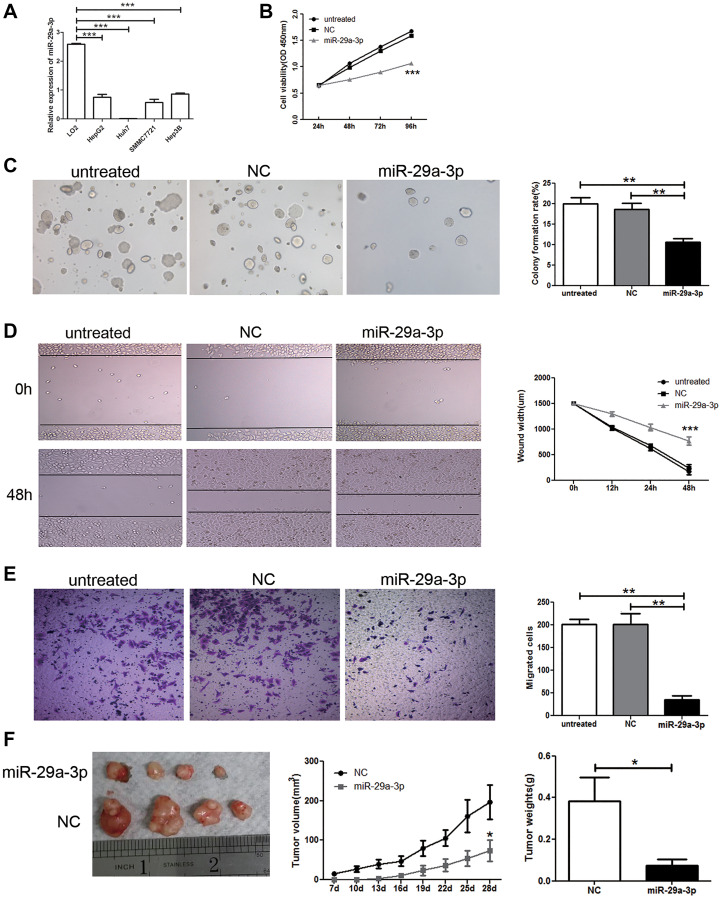
Overexpression of miR-29a-3p inhibited cancer cell growth and migration *in vitro* and *in vivo*. (**A**) QRT-PCR analysis of miR-29a-3p expression in normal human hepatic cell line (LO2) and HCC cells lines (SMMC-7721, Hep3B, HepG2, and Huh 7). (**B**) Proliferation ability test by CCK8 assay of HepG2 cells after transfection with miR-29a-3p mimics, negative control (NC) or no transfection (untreated). (**C**) Colony formation assay and statistical results in HepG2 cells after transfection with miR-29a-3p mimics, NC or untreated. (**D**) Wound healing assay of HepG2 cells after transfection with the miR-29a-3p mimics, NC or untreated. (**E**) Transwell migration assay of HepG2 cells after transfection with the miR-29a-3p mimics, NC or untreated. (**F**) Functional test of miR-29a-3p *in vivo* and statistical results. (^*^ *P* < 0.05, ^**^ *P* < 0.01, ^***^ *P* < 0.001).

